# Single-Camera Multi-View 6DoF pose estimation for robotic grasping

**DOI:** 10.3389/fnbot.2023.1136882

**Published:** 2023-06-13

**Authors:** Shuangjie Yuan, Zhenpeng Ge, Lu Yang

**Affiliations:** Fundamental Research Center, School of Automation Engineering, University of Electronic Science and Technology of China, Chengdu, China

**Keywords:** 6DoF pose estimation, multi-view, monocular motion, industrial robots, deep learning in robotic grasping

## Abstract

Accurately estimating the 6DoF pose of objects during robot grasping is a common problem in robotics. However, the accuracy of the estimated pose can be compromised during or after grasping the object when the gripper collides with other parts or occludes the view. Many approaches to improving pose estimation involve using multi-view methods that capture RGB images from multiple cameras and fuse the data. While effective, these methods can be complex and costly to implement. In this paper, we present a Single-Camera Multi-View (SCMV) method that utilizes just one fixed monocular camera and the initiative motion of robotic manipulator to capture multi-view RGB image sequences. Our method achieves more accurate 6DoF pose estimation results. We further create a new T-LESS-GRASP-MV dataset specifically for validating the robustness of our approach. Experiments show that the proposed approach outperforms many other public algorithms by a large margin. Quantitative experiments on a real robot manipulator demonstrate the high pose estimation accuracy of our method. Finally, the robustness of the proposed approach is demonstrated by successfully completing an assembly task on a real robot platform, achieving an assembly success rate of 80%.

## 1. Introduction

**Figure 1:**
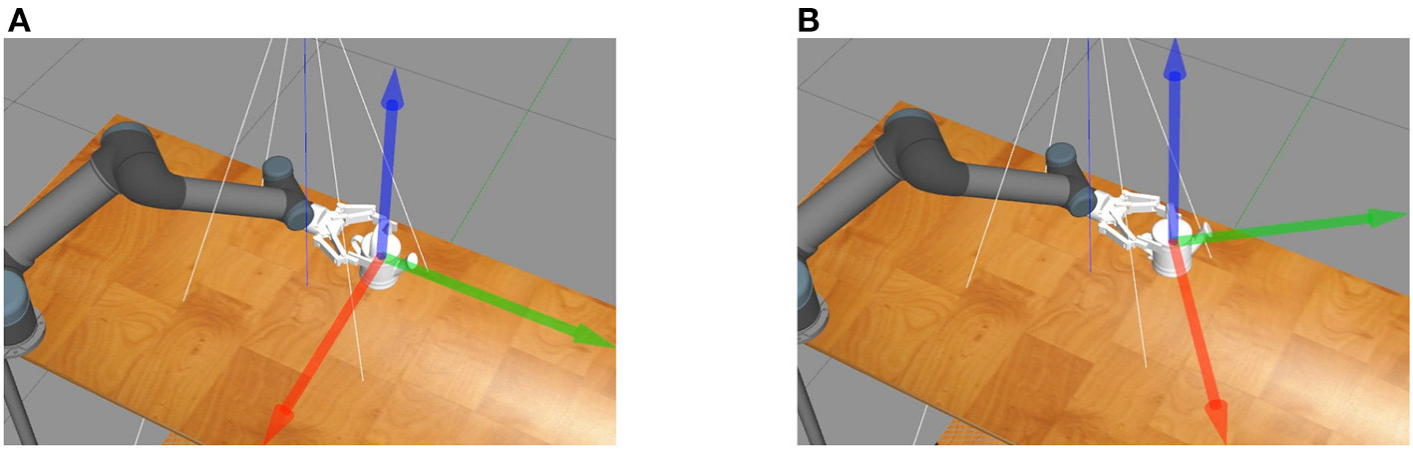
The collision between the gripper and assembly parts. Three arrows represent the pose of the part. **(A)** The pose of the part before grasping is parallel to the edge of the table. **(B)** A collision occurs while grasping the part resulting in a change of the part's pose.

This paper focuses on the challenge of 6DoF pose estimation while the robot grasping objects, as the estimated pose is inaccurate due to the collision of the gripper with assembly parts and the gripper's self-occlusion. In the process of grasping assembly parts, the gripper will collide with the assembly parts, which causes previously estimated part pose to be inaccurate. As the Figure 1 shows, the three arrows represent the direction of the base coordinate system of the workpiece in relation to the world coordinate system, and indicate the pose of the part. The pose of the part before grasping is shown in Figure 1A, with its pose parallel to the edge of the table. After the first grasp by the robotic arm, as shown in Figure 1B, the part's pose changes due to the collision with the end effector of the manipulator. Therefore, it is necessary to re-estimate the pose of the part.

To solve this problem, this paper proposes a Single-Camera Multi-View (SCMV) pose estimation method. In contrast to existing mainstream methods or applications using multiple cameras in industrial manufacturing, this method utilizes the initiative motion of the robotic manipulator to obtain multi-view information of assembly parts under a fixed monocular camera. And the SCMV pose estimation method can be directly implemented in a variety of applications, particularly in peg-in-hole assembly, due to its efficiency for various industrial parts.

The most significant contributions to this paper are:

- Build the optimization model for the SCMV 6DoF pose estimation problem.
- Propose a 6DoF pose estimation method which utilizes the initiative motion of robotic manipulator to obtain multi-view information of parts under one monocular camera.
- Refine the multi-view image sequence for the SCMV pose estimation method to address issues of pose estimation inaccuracy due to the gripper colliding with and occluding the object during grasping.

In comparison with other public algorithms like Cosypose (Labbé et al., 2020), Implicit (Sundermeyer et al., 2018), and Pix2pose (Park et al., 2019), the proposed method achieves the optimal effect. Also, on the real robot manipulator platform, the proposed SCMV algorithm is experimentally validated.

This paper is grouped as follows: Section 2 introduces the related works, Section 3 introduce the SCMV 6Dof pose estimation method and its refinement, Section 4 display the experiments and the analysis in simulation cases and on the real robot manipulator platform, and the conclusion is given in Section 5.

## 2. Related works

The intelligent manufacturing has become the trend of industrial manufacturing as the artificial intelligence and other technology developed. Germany proposed the “Industry 4.0” (Lasi et al., 2014), which was launched at the Hannover Messe, in 2013. It focuses on intelligent production to achieve smart factories, intelligent production, and intelligent logistics. General Electric introduced the concept of “Industrial Internet” (Agarwal and Brem, 2015) similar to the “Industry 4.0” in 2013, which connected the modern Internet with industrial machines, giving them intelligence and redefining industrial manufacturing. The core of “Industry 4.0” and “Industrial Internet” is to use the Internet, artificial intelligence, and other innovative technologies in industrial manufacturing to promote the transformation of traditional manufacturing industries to automated intelligent manufacturing.

6D object pose estimation consists primarily of two methods divided by estimation types: correspondence-based methods and template-based methods.

The correspondence-based methods involve locating the correspondence between the input data and the complete 3D point cloud of the existing object, which is typically the known CAD model. When the input is a 2D image, such 2D image-based methods are mainly to estimate the pose of the objects with rich texture. To get the 2D feature points, these 2D feature descriptors such as SIFT (Lowe, 1999), FAST (Rosten and Drummond, 2005), SURF (Bay et al., 2006), and ORB (Rublee et al., 2011), etc., are commonly utilized and efficient. After getting the correspondence between 2D pixels and 3D points of the existing 3D model, the pose can be obtained by Perspective-n-Point (PnP) (Lepetit et al., 2009) method, which is similar to the keyframe-based SLAM approach (Mur-Artal et al., 2015) proposed by Mur-Artal et al. Similarly, in the field of SLAM, there are many methods that fuse multi-source information to reduce errors. Specifically, Munoz-Salinas and Medina-Carnicer (2020) propose a multi-scale strategy to speed up marker detection in video sequences by selecting the most suitable markers. And Poulose and Han (2019) proposed a hybrid system to reduce IMU sensor errors by using smartphone camera pose and heading information, resulting in improved accuracy. Cosypose (Labbé et al., 2020) obtains multi-view information from cameras. In addition to conventional feature descriptors, deep learning-based feature descriptors have appeared. PVNet (Peng et al., 2019) predicted 2D feature points and then found the corresponding 2D-3D correspondences to estimate the 6D object pose. Besides explicitly finding the correspondences between feature points, many deep learning-based methods implicitly predict the projection position corresponces between 3D points on 2D images. Since 3D feature points on objects cannot be directly selected, Rad et al. proposed BB8 (Rad and Lepetit, 2017) method which predicted the projection of 8 vertices of the minimum 3D bounding box of objects on 2D images. Since the projected points of the bounding box may be located outside the image, the Dpod proposed by Zakharov et al. (2019) predicted all the correspondences between 3D points and 2D points in the object area on the 2D image. Similarly, Park et al. proposed the pix2pose method of regressing 3D coordinates of objects from 2D images using 3D CAD models without textures.

As for 3D point cloud input, the correspondence-based methods usually utilize 3D feature descriptors to find the correspondences between two point clouds. The 3D–3D correspondences are directly used to get the 6D object pose. In conventional approaches, these 3D local feature descriptors, such as Spin Images (Johnson, 1997), 3D Shape Context (Frome et al., 2004), FPFH (Rusu et al., 2009), CVFH (Aldoma et al., 2011), SHOT (Salti et al., 2014), etc., are used to obtain correspondences between the local 3D point cloud and the complete point cloud of the object.

To deal with objects with weakly textured or untextured images, template-based 6D object pose estimation methods are more suitable. The representative method of template-based 6D pose estimation from 2D images is the LineMode (Hinterstoisser et al., 2012) method, which finds the most similar template image by comparing the gradient information between the observed 2D images and the template 2D images. The LineMode method can also combine the normal vector of the depth map to reduce the error. In addition to finding the most similar template image explicitly, there are also ways to find the most similar template implicitly. The perspective approach is Implicit (Sundermeyer et al., 2018). The Implicit learns the object's pose by using an enhanced self-encoder, which can effectively handle ambiguous pose estimation with occlusion. Some methods reconstruct the 6D pose of the target object directly from the image, whose process can be regarded as finding the image most similar to the current input image from the trained labeled images and outputting its 6D pose. These methods directly obtain the transformation from the input image to the pose parameter space and are easy to apply within the target detection framework. There are numerous such methods, the representatives of which are PoseCNN (Xiang et al., 2017), SSD6D (Kehl et al., 2017), and Deep-6DPose (Do et al., 2018). Another type of method, such as NOCS (Wang et al., 2019), generates implicit correspondences for a class of objects; these methods are also template-based.

To get the 6D object pose, traditional registration methods usually find the 6D transformation that best aligns the partial point cloud to the full point cloud of the CAD model. The methods based on 3D point clouds are mainly global registration methods, such as Super 4PCS (Mellado et al., 2014) and GO-ICP (Yang et al., 2015). And the predicted pose can be optimized by icp methods. Some deep learning methods for registering and aligning two point clouds have also emerged, including PCRNet (Sarode et al., 2019), DCP (Wang and Solomon, 2019), and PointNetLK (Aoki et al., 2019). During registration, combining multiple views can make the input data more complete, or a complete object can be projected from multiple views to obtain multiple single point clouds to help registration.

In addition, among robotic assembly studies, there are many assembly scenarios, such as peg-in-hole assembly (Pauli et al., 2001; Yang et al., 2020), chute assembly (Peternel et al., 2018), bolt assembly (Laursen et al., 2015), etc. The peg-in-hole assembly is the most common of all assembly tasks and the one most commonly studied. There are a variety of assembly methods for these assembly scenarios, including programming based control methods, demonstration methods, vision feedback based methods, force feedback based methods, and multi-method fusion methods.

Inspired by the papers discussed above, a method using a fixed monocular camera to obtain multi-view RGB images for pose estimation is proposed. Unlike other state-of-the-art methods that typically require multiple cameras or moving a monocular camera to obtain multi-view information, the proposed method utilizes the initiative motion of the robotic manipulator which means the robot manipulator can reach any point in its workspace to obtain multi-view information with a fixed monocular camera. Additionally, the method optimizes the SCMV algorithm proposed in this paper using the minimum reprojection error.

## 3. Methods

In this section, we propose our Single-Camera Multi-View (SCMV) Pose Estimation Method. Firstly, we introduce the modeling of SCMV Pose Estimation. Then, we refine the multi-view image sequence for the SCMV pose estimation method to reduce the estimation error.

### 3.1. Single-Camera Multi-View (SCMV) 6DoF pose estimation

**Figure 2:**
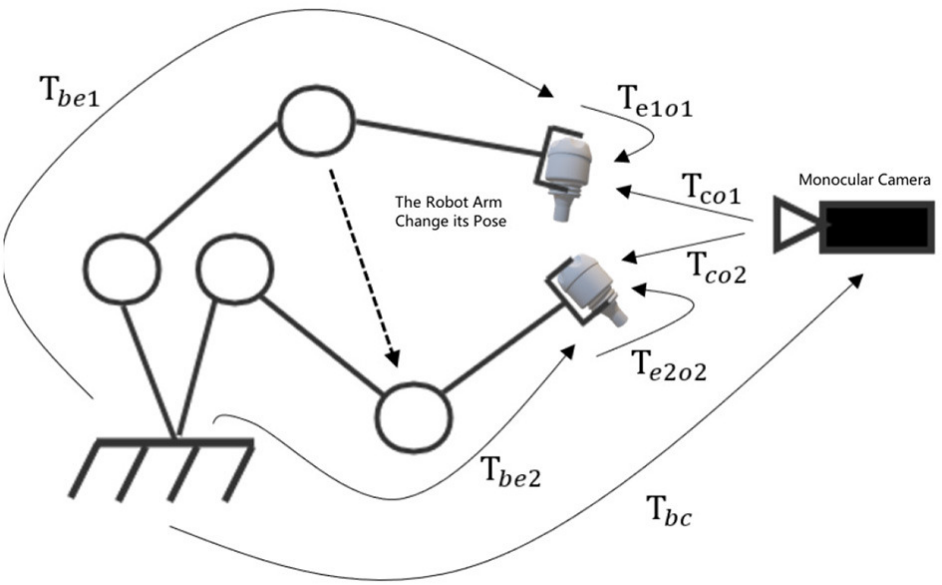
The coordinate transformation for multiple-views from a monocular camera.

When estimating the pose of an object, if we use cameras to collect different views of the same object, we can fuse the results of multiple views. For each view, we calculate the minimum reprojection error. By optimizing using the minimum reprojection error, a more precise pose estimation can be obtained, which is also the BA(Bundle Adjustment) problem in SLAM. Thus, the object-level BA algorithm is utilized to estimate the pose of multiple objects, thereby obtaining improved results. There is only one camera in our method, and its position is fixed. Due to the initiative motion of robotic arm, we can alter its pose to alter the pose of the end-effector, and we can obtain multiple views of the assembly parts with just one monocular camera. The BA method's concept of minimum reprojection error is used to construct a non-linear optimization model. Optimize multiple low-precision single-view pose estimation results for higher accuracy by solving the optimization model. The coordinate transformation for multiple-views from a monocular camera is shown in Figure 2.

A new view is obtained for each initiative motion of the robot arm. Using the single-view pose estimation algorithm for pose estimation at each view, the pose at each single view can be determined. Due to the fact that the coordinate transformation of the end-effector relative to the camera can be measured and calculated, the coordinate transformation between multiple views can be determined, thereby making multi-view information fusion accessible. The coordinate transformation of the assembly part relative to the end of the end-effecto is *T*_*eo*_ ∈ *SE*(3). Changing the pose of the robot arm can change the pose of assembly parts. We can get a range of views *V*_1_, *V*_2_, *V*_3_ *of*, …*V*_*n*_ of the assembly part with the fixed monocular camera, where *V*_*i*_ is the *i*th measurement of the part. Assume that the true pose of the part in the camera coordinate system is *T*_*c*_*o*__*i*__. Using the deep-learning based single-view pose estimation algorithm under the *V*_*i*_, the estimated pose in the camera coordinate system is ${\hat{T}}_{co_{i}}$, and the measured joint angle this time is ${\hat{\theta }}_{n}$. Using the concept of minimum reprojection error, we can get the optimal objective function shown in Equation (1).


(1) $$\begin{array}{l} \underset{T_{co_{i}}}{\min}\overset{n}{\underset{i=1}{\sum }}\underset{x\in S_{o}}{\sum }||\pi \left(T_{co_{i}}x\right)-\pi \left({\hat{T}}_{co_{i}}x\right)|{|}^{2} \end{array}$$

In Formula (1), π(·) is the camera projection function which projecting three-dimensional points in space onto a two-dimensional picture. This function is a non-linear transformation. *S*_*o*_ a collection of point clouds on the object model.

When the pose of the robot arm is changed, the coordinate transformation of the part can be decomposed and transformed according to the coordinate transformation principle, as shown in Equation (2).


(2) $$\begin{array}{l} T_{co_{i}}=T_{ce_{i}}T_{e_{i}o_{i}} \end{array}$$

In order to simplify the objective function, the connection between different views must be established during the robot arm's grasping of the assembly parts, along with the following two assumptions:

1. The moving accuracy of the arm is very high.
2. No more sliding after the robot arm grasps the object.

For Assumption 1., currently, the repeatability of collaborative robot arms is typically under 0.2 mm. For certain simple, flexible assembly tasks, millimeter-level repeatability meets the required level of precision. Consequently, the hypothesis is reasonable. Based on this assumption, it can be assumed that the end-effector's measured pose is its real pose. The measured pose can be calculated by the forward kinematics of the robot arm, as shown in Equation (3).


(3) $$\begin{array}{l} T_{be_{i}}={\hat{T}}_{be_{i}}=T_{arm}\left({\hat{\theta }}_{n}\right) \end{array}$$

For Assumptions 2, this paper is only for assembly tasks of rigid parts. There is no deformation of the gripper while grasping. Typically, anti-slip material is attached to the gripper's end. When the component is grasped, there will be no sliding. So this assumption is also reasonable. Based on this assumption, it can be assumed that the pose of the object is fixed relative to the end-effector when the part is grasped. This means that the pose of the assembly part is constant relative to the end-effector of the arm from different perspectives, as shown in Equation (4).


(4) $$\begin{array}{l} T_{eo}=T_{e_{i}o_{i}} \end{array}$$

Final optimization function is obtained based on Equations (1), (2), (3), and (4), as shown in Equation (5).


(5) $$\begin{array}{l} \underset{T_{eo}}{\min}\overset{n}{\underset{i=1}{\sum }}\underset{x\in S_{o}}{\sum }||\pi \left(T_{cb}{\hat{T}}_{be_{i}}T_{eo}x\right)-\pi \left({\hat{T}}_{co_{i}}x\right)|{|}^{2} \end{array}$$

The optimization objective of this objective function is the coordinate transformation of the part to the end-effector, which is a non-linear optimization problem. Methods such as the Levenberg-Marquardt method or graph optimization can be used to get the estimation of the assembly part's pose ${\hat{T}}_{eo}$ in the end-effector coordinate system of the robot arm. This allows us to correct the pose estimation of the assembly part in the camera coordinate system, as shown in Equation (6).


(6) $$\begin{array}{l} {\hat{T}}_{co}=T_{cb}{\hat{T}}_{be}{\hat{T}}_{eo}=T_{cb}T_{arm}\left({\hat{\theta }}_{n}\right){\hat{T}}_{eo} \end{array}$$

In the monocular multi-view pose estimation model, the assembly part pose estimation problem in the camera coordinate system is transformed into the end-effector coordinate system, which allows us to get multi-view information with just one fixed camera. By fusing multi-view information, multiple low-precision pose estimation results can be further optimized to obtain high-precision pose estimation results.

Pseudo-code flow of SCMV Pose Estimation algorithm as shown in Algorithm 1.

**Algorithm 1:** SCMV 6DoF pose estimation algorithm.

**Output**: *T*_*eo*_
1. Get a range of views *V*_1_, *V*_2_, *V*_3_ *of*, ⋯*V*_*n*_ of the assembly part with the fixed monocular camera;
2. For each view, use the single-view pose estimation algorithm,and get the estimated pose in the camera coordinate system ${\hat{T}}_{co_{i}}$, and the joint angle ${\hat{\theta }}_{n}$
3. Calculate coordinate transformation of the part *T*_*c*_*o*__*i*__ = *T*_*c*_*e*__*i*__*T*_*e*_*i*_*o*_*i*__
4. Using the concept of minimum reprojection error, and the previous steps, get the final optimization function $\underset{T_{eo}}{\min}\overset{n}{\underset{i=1}{\sum }}\underset{x\in S_{o}}{\sum }||\pi \left(T_{cb}{\hat{T}}_{be_{i}}T_{eo}x\right)-\pi \left({\hat{T}}_{co_{i}}x\right)|{|}^{2}$
5. Use G2O to solve the final optimization function ${\hat{T}}_{co}=T_{cb}{\hat{T}}_{be}{\hat{T}}_{eo}=T_{cb}T_{arm}\left({\hat{\theta }}_{n}\right){\hat{T}}_{eo}$

### 3.2. Refined SCMV 6DoF pose estimation

It is assumed that in the optimal model of SCMV pose estimation, the errors of estimation from different views are independent and identically distributed. On the basis of this assumption, we can utilize the optimal method for fusing the multiple-view data, and get a lower estimation error. In fact, the estimation error for assembly parts is not the same from different views. Some views have small estimation errors, while others have larger estimation errors. So it is necessary to filter these views.

During the active vision pose estimation, the estimated pose is inaccurate due to the gripper's self-occlusion. In addition, the results of self-occlusion vary depending on the different views. Pose estimation of assembly parts from a single camera is always dependent on the image features of the parts, regardless of the method used, as traditional methods require the detection of image key points before pose estimation, whereas deep learning methods typically use CNN to extract features before pose estimation. The self-occlusion of the gripper may mask some key feature points of the assembly parts, thereby affecting the precision of the estimation. Consequently, the estimation errors from various views are uncertain. Choose one T-LESS part to illustrate this situation by rendering diagrams from different views under different grasping poses in the simulation environment, as shown in Figure 2.

**Figure 3:**
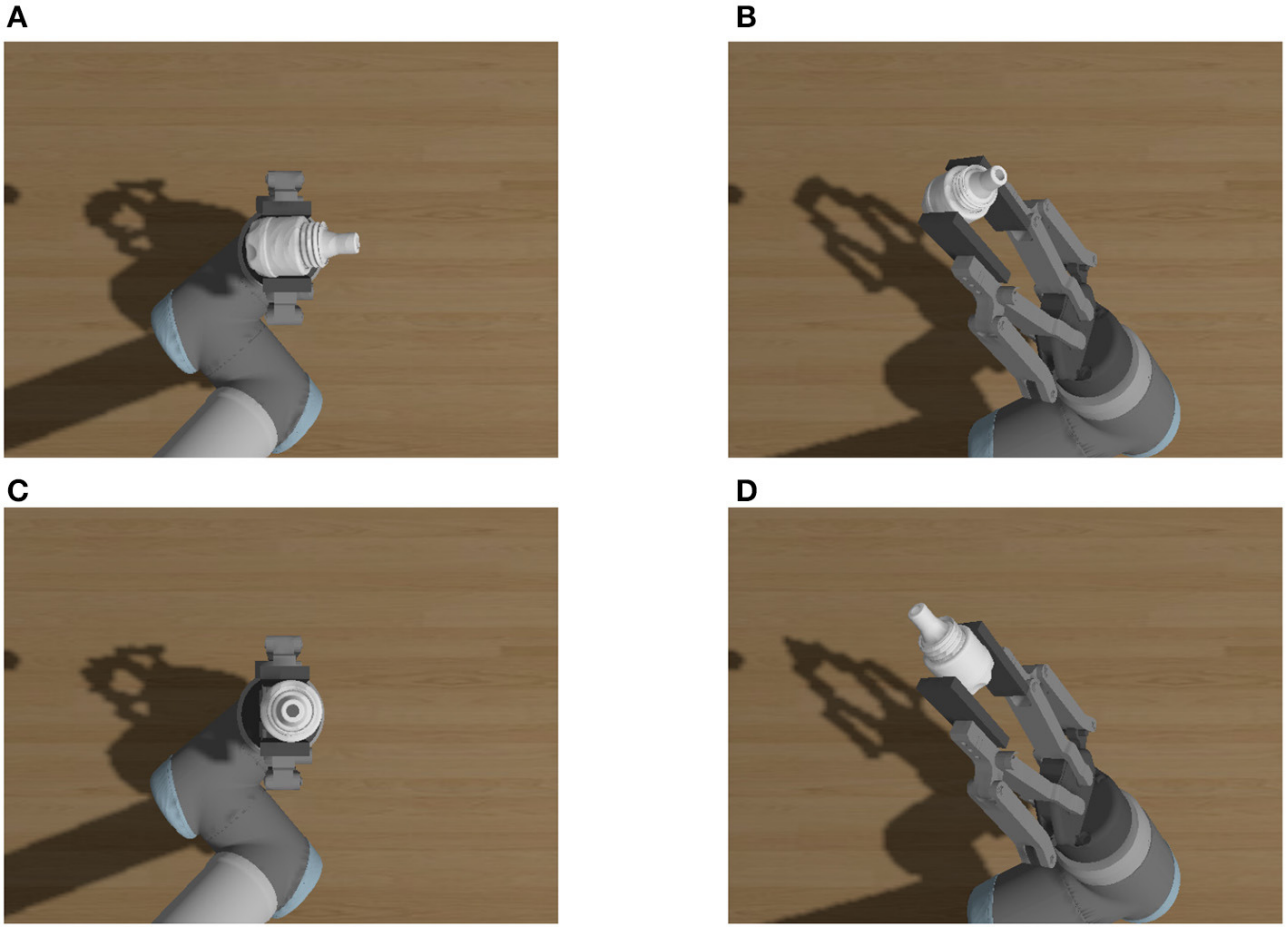
Occlusion and self-occlusion from different views. **(A)** View 2–4 from pose1. **(B)** View 4–0 from pose1. **(C)** View 2–4 from pose3. **(D)** View 4–0 from pose2.

It is not difficult to conclude that occlusion and self-occlusion situations are complex, and that the problems of gripper's occlusion and self-occlusion must be considered simultaneously. Figures 3A, B illustrate the ideal grasping situation of the end effector of the robotic manipulator. Figure 3D has a smaller gripper occlusion than Figure 3C, but the self-occlusion is the different. The occlusion is related to the shape of the assembly parts and the gripper's pose. Therefore, it is practical to manually select the best estimation view. In the process of single-camera multi-view optimization, using views with high error to optimize the pose estimation will reduce the accuracy of optimization. If a single view sample produces inaccurate estimation results due to occlusion or self-occlusion, such view samples may produce worse optimization results than the single-view estimation.

We refine the multi-views image sequence in the SCMV 6DoF Pose Estimation Method to solve this problem. As the errors of pose estimation from different views are different, all views can be divided into two categories based on the RANSAC (Fischler and Bolles, 1981) algorithm: those with small estimation errors and those with larger estimation errors. In fact, we are unable to directly calculate the view errors because we do not know the real pose of the assembly parts. It can be assumed that most of the results estimated from the data are within the error range, while a few of the results are with high errors. Based on it, it is necessary to refine the multi-view image sequence to filter the subset of views with higher consistency automatically.

The Refined SCMV 6DoF Pose Estimation algorithm can be divided into three steps: Multi Views Sampling, refining multi-views image sequence and Optimization:

1. Multi-View Sampling
  Using the initiative of the robot arm, sample from multiple views and get multi-view data collection *S*_*v*_ of the grasped assembly part.
2. Refining Multi-views Image Sequence Random sample set *S*_*v*_ Calculate the reprojection error and construct a consistent set $S_{v}^{\prime }\subseteq S_{v}$. The consistency set *S*_best_ with the smallest error is obtained through iterations.
3. Optimization
  Using a set of consistent view subsets Sbest obtained in the preceding step, we optimally solve the G2O (Kümmerle et al., 2011) optimized monocular multi-view minimum reprojection error model to obtain the results.

Pseudo-code flow of Refined SCMV Pose Estimation algorithm as shown in Algorithm 2:

**Algorithm 2:**
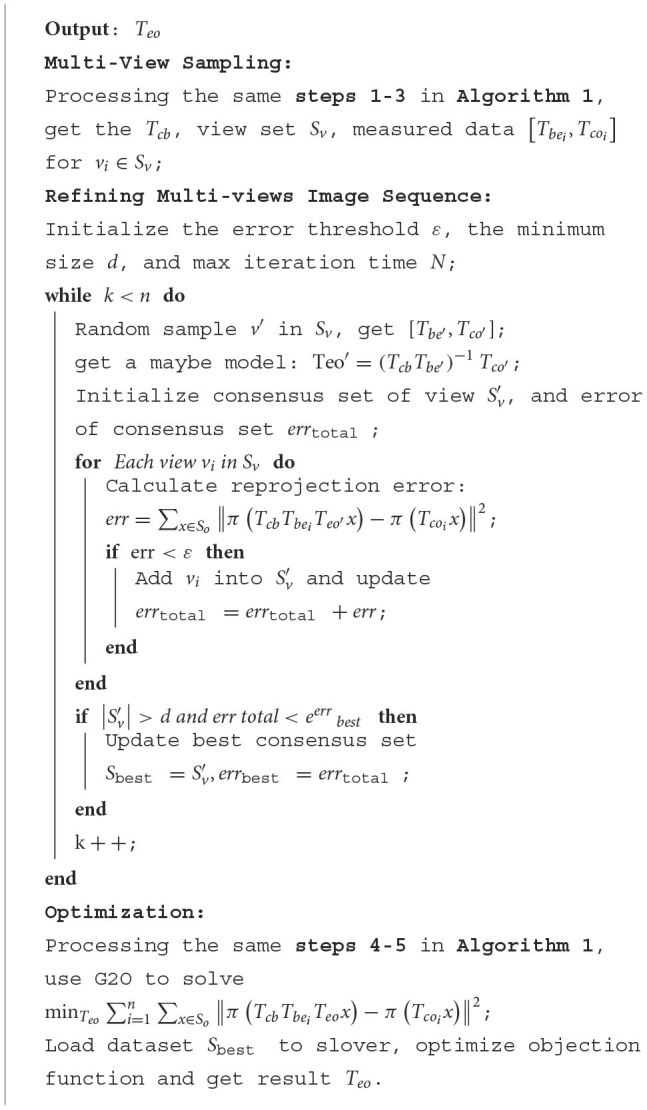
Refined SCMV 6DoF pose estimation algorithm.

## 4. Experiments

In the section, we verify the effectiveness of the proposed method and evaluate its application of peg-in-hole assembly.

### 4.1. Test datasets and evaluation indicators

**Figure 4:**
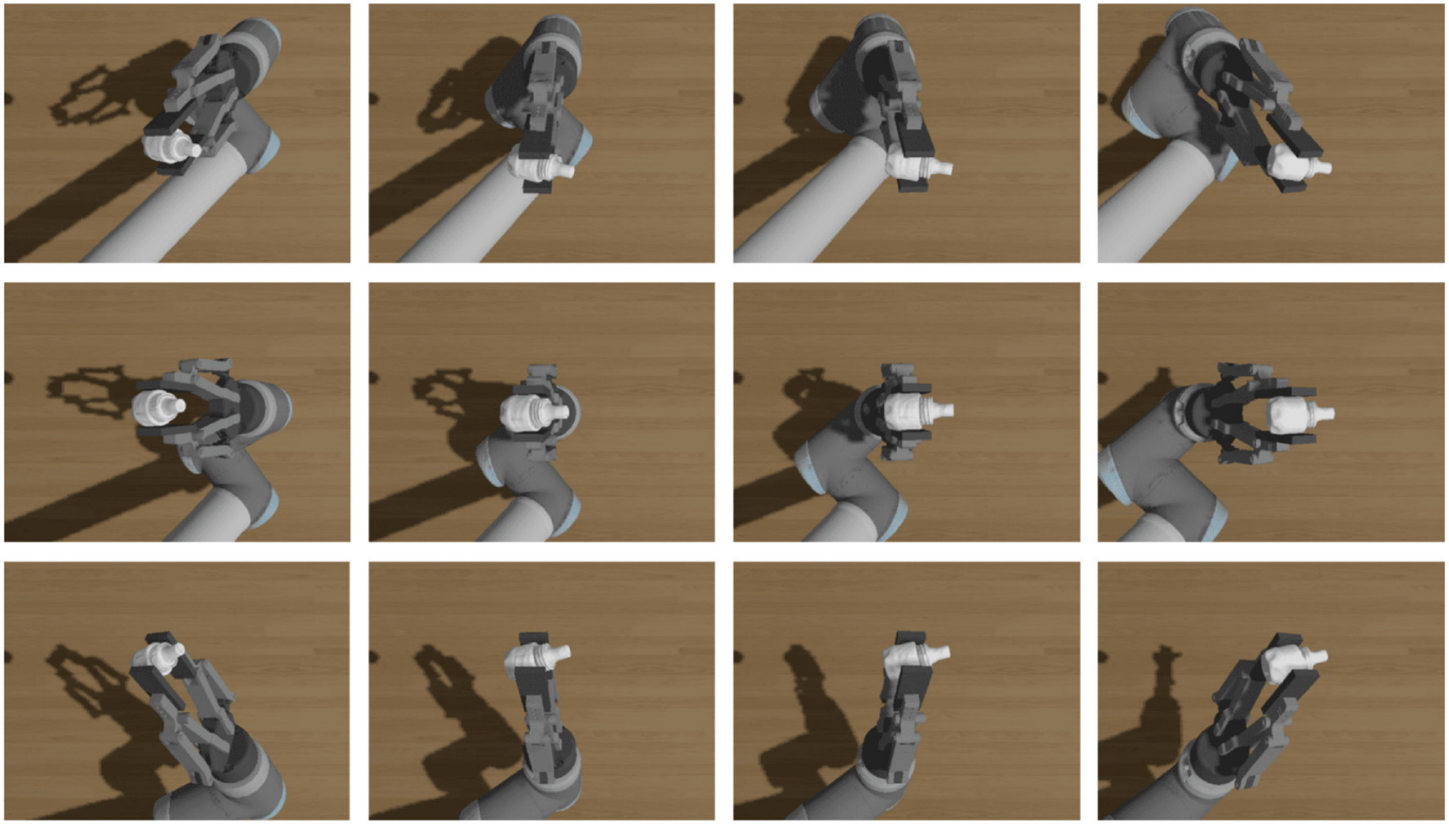
The multi-view diagram of Part 1.

We first design a simulation experiments with the benchmark we built, in order to further study the influence of these factors on the final results of multi-view-based pose estimation. A dataset T-LESS-GRASP-MV is constructed for the optimization of robotic arm SCMV 6DoF pose estimation based on the Gazebo robot simulation platform using the T-LESS (Hodan et al., 2017) industrial parts dataset. The multi-view diagram of Part 1 of T-LESS is shown in Figure 4.

The T-LESS-GRASP-MV dataset consists of 10 test assembly parts and contains a total of 1,350 single-camera and multi-view pictures of the parts, which can be used for multi-view pose estimation evaluation. In order to evaluate the efficacy of the SCMV algorithm, the part pose estimation errors are divided into two indicators: translation error and rotation error, respectively. These two indicators are intuitive and can be used to evaluate the accuracy of pose estimation in assembly tasks.

The real position of a part in a assembly grasping test is [*t, R*]. The estimated position is $\left[\hat{t},\hat{R}\right]$. The calculation of the translation error *dt* is shown in Equation (7).


(7) $$\begin{array}{l} dt=||\hat{t}-t||=\sqrt{\Delta x^{2}+\Delta y^{2}+\Delta z^{2}} \end{array}$$

Since the rotation belongs to the SO(3) group, the rotation error cannot be calculated by directly subtracting the rotation matrix. We first calculate the rotation increment Δ*R* in matrix space using matrix multiplication, as shown in Equation (8).


(8) $$\begin{array}{l} \Delta R={\hat{R}}^{-1}R \end{array}$$

Transform the Δ*R* into euler angles (Δα, Δβ, Δγ). Its vector module is used as the rotation error *dr*, as shown in Equation (9).


(9) $$\begin{array}{l} dr=||\mathrm{rpy}\left(\Delta R\right)||=\sqrt{\Delta \alpha^{2}+\Delta \beta^{2}+\Delta \gamma^{2}} \end{array}$$

Meanwhile, the T-LESS dataset contains multiple rotationally symmetric objects, and the rotation error of such objects must account for their rotationally symmetric properties, whose calculation of rotation error is shown in Equation (10),


(10) $$\begin{array}{l} dr=\underset{R_{S}\in S}{\min}||\mathrm{rpy}\left({\hat{R}}^{-1}RR_{S}\right)|| \end{array}$$

where *R*_*S*_ is the symmetric rotation matrix of the part, *S* is the collection of all the *R*_*S*_ for this part. If the object is a continuous rotationally symmetric object, the set *S* of *R*_*S*_ must be discretized.

### 4.2. Comparative experiments with related works

This section compares the SCMV Pose Estimation method with several open-source object pose estimation algorithms on the T-LESS-GRASP-MV test dataset, utilizing translation error and rotation error as indicators. Several methods that have reached SOTA in recent years are compared. Implicit (Sundermeyer et al., 2018) learns the object's pose by using an enhanced self-encoder, which can effectively handle ambiguous pose estimation with occlusion. Pix2pose (Park et al., 2019) regresses the pose of an object by predicting the 3D position of each pixel. Cosypose (Labbé et al., 2020) is based on the concept of DeepIM (Li et al., 2020), and by utilizing rotation parameterization and other methods, we can obtain better pose estimation results. In this section, for the single-view pose estimation method, the pose is estimated and the estimation error is computed for each of the 45 grasping pose views using the corresponding algorithm. The average error for all views is the error for this set of views. The estimation error results of the different methods are shown in Table 1. The “Part 1 to 23” label in the table, these refer to the different assembly parts used in our experiments. The dt(mm) and dr(rad) represent the translation error *dt* and the rotation error *dr*.

**Table 1:** Experimental results on T-LESS-GRASP-MV dataset.

	**Implicit (Sundermeyer et al.**, **2018****)**		**Pix2pose (Park et al.**, **2019****)**		**Cosypose (Labbé et al.**, **2020****)**		**SCMV**	
	**dt (mm)**	**dr (rad)**	**dt (mm)**	**dr (rad)**	**dt (mm)**	**dr (rad)**	**dt (mm)**	**dr (rad)**
Part 1	6.183	0.182	5.173	0.172	2.816	0.077	**0.564**	**0.021**
Part 4	5.278	0.113	4.762	0.108	2.362	0.054	**0.908**	**0.035**
Part 5	4.379	0.097	4.254	0.075	2.183	0.042	**0.393**	**0.012**
Part 6	4.294	0.115	4.189	0.129	2.335	0.068	**0.418**	**0.017**
Part 11	5.936	0.172	5.972	0.154	3.399	0.098	**0.898**	**0.03**
Part 13	4.528	0.095	4.183	0.089	1.987	0.048	**0.588**	**0.012**
Part 19	4.221	0.114	4.319	0.084	2.195	0.038	**1.146**	**0.011**
Part 20	5.728	0.118	5.214	0.103	2.427	0.047	**1.013**	**0.014**
Part 21	6.462	0.148	6.172	0.136	3.224	0.065	**1.501**	**0.037**
Part 23	6.192	0.094	5.923	0.097	3.337	0.064	**0.905**	**0.032**

The bold values indicate that the data was obtained by the SCMV 6Dof Pose Estimation algorithm proposed in this paper, and the error is smaller than other public algorithms.

From the table, it is easy to find that the SCMV pose estimation method achieves the best estimation accuracy compared to other methods for different shapes of parts with different grasping poses. The percentage to which our method reduces the error compared to the SOTA method can be calculated according to the following Equation (11):


(11) $$\{\begin{array}{l} \frac{\left|d_{r_{SCMV}}-\right|d_{r_{\text{ Other }}}|}{\left|d_{r_{\text{Other}}}\right|}*100\%\\ \frac{\left|d_{t_{SCMV}}-\right|d_{t_{\text{ Other }}}|}{\left|d_{t_{\text{Other}}}\right|}*100\% \end{array}$$

Compared with Implicit (Sundermeyer et al., 2018), the SCMV pose estimation method reduced the average translation error by 84.34% and the average rotation error by 82.25%. Moreover, the average translation and rotation errors are reduced by 68.27 and 63.33%, respectively, when compared to the Cosypose (Labbé et al., 2020) algorithm. This demonstrates that the multi-view approach effectively combines data from multiple views. The single-camera multi-view method based on minimum reprojection error optimization is superior to the simple average strategy method because the optimization method uses the constraints of the camera projection relationship to optimize, uses geometric priori information as opposed to the simple averaging strategy, and utilizes the RANSAC method to filter out the views with high estimation errors. Therefore, the SCMV pose estimation method can improve the accuracy and robustness of pose estimation results.

### 4.3. The influence of the views num on the SCMV pose estimation algorithm

This section shows how the number of views affects the SCMV pose estimation algorithm. The multi-view method is significantly better than the single-view method. However, in the process of collecting multiple views with the robot arm, each view requires a certain amount of time, and the number of views is proportional to the sampling time. Therefore, the number of views must be weighed against the sampling time and precision. In order to determine the relationship between the number of views and the accuracy of pose estimation, we ran tests on the T-LESS-GRASP-MV dataset, where each set of multi-view data contains 45 views, sampled multiple times with different numbers of views, and then used the SCMV pose estimation method to estimate the corresponding part pose and calculate the translation error and rotation error, respectively. Estimation errors with different number of views are shown in Table 2.

**Table 2:** Estimation errors with different number of views.

**Views number**	**2**	**4**	**6**	**8**	**10**	**12**	**14**	**20**	**30**	**40**
dt(mm)	2.544	2.213	1.748	1.262	1.016	0.885	0.832	0.840	0.817	0.838
dr(rad)	0.052	0.042	0.034	0.023	0.021	0.020	0.021	0.022	0.021	0.021

**Figure 5:**
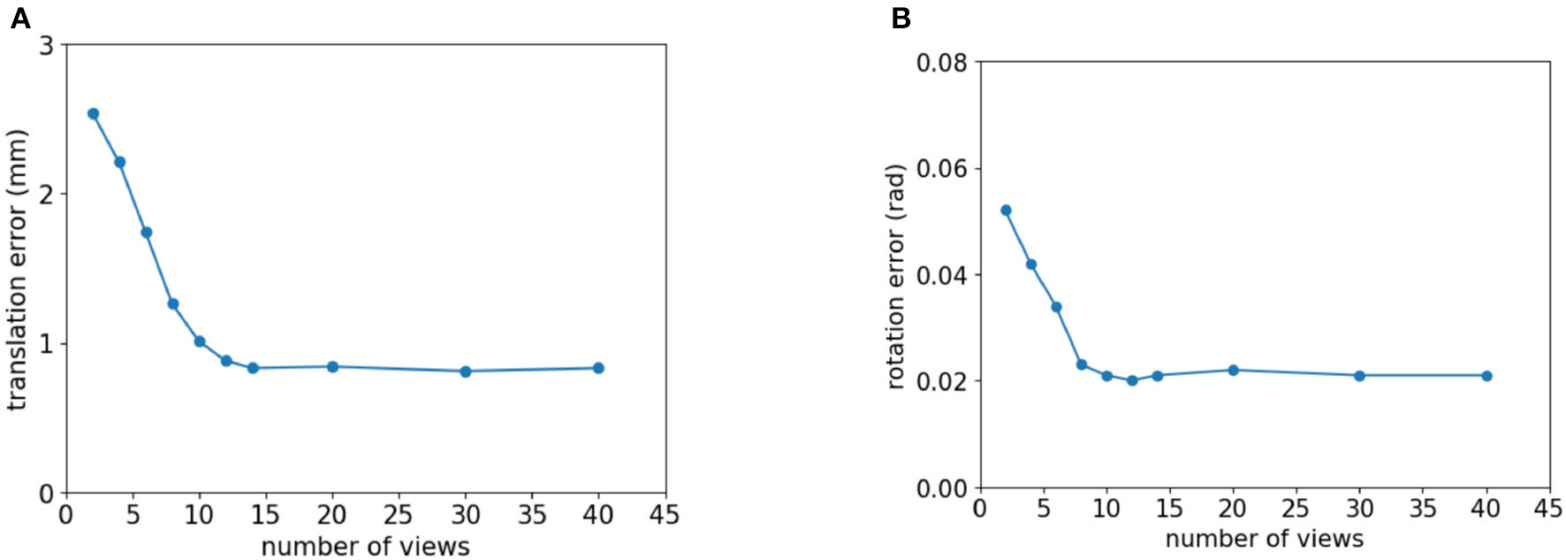
The relationship between the number of views and the error of pose estimation whose view number is view 4–0 from pose1. **(A)** Translation error. **(B)** Rotation error.

According to Table 2, when the number of views exceeds 14, the average translation error and the average rotation error remain relatively stable and no longer decrease significantly as the number of views increases. As shown in Figure 5, the relationship between the number of views and the estimation accuracy of the algorithm can be depicted as a line graph.

It is evident from Figure 5 that the translation error and rotation error of the pose estimation have a negative relationship with the number of views but are not linearly related to one another. When the number of views is small, the translational and rotational errors in the positional estimation decrease considerably as the number of views increases. Translation and rotation errors no longer decrease significantly as the number of views reaches a certain threshold because the estimation result from a single-view contains an error. When a view is added to an optimization model, the amount of information increases while the uncertainty error also increases. Therefore, the accuracy of SCMV pose estimation does not always improve as the number of views increases.

### 4.4. SCMV pose estimation on the real robotic manipulator

**Figure 6:**
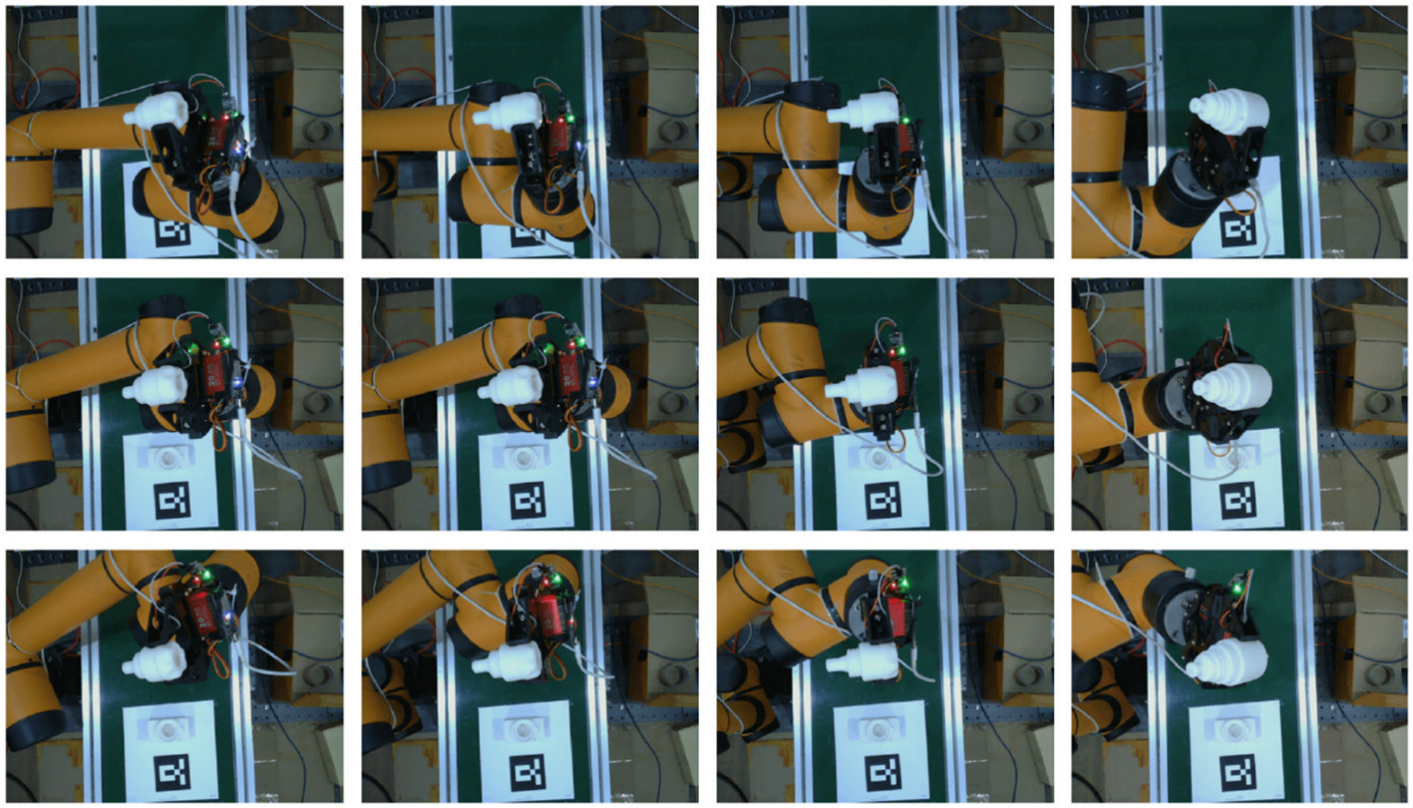
Multi-view images captured in a real environment (partial views).

In this section, the SCMV algorithm was applied to pose re-estimation on real robotic manipulator. The AUBO-i5 Collaborative Robot and a two-finger parallel gripper were used in the experiment, and RGB images were captured by the Realsense L515 camera. First, the objects were placed without obstruction on the workbench, and then the Cosypose method was used for initial pose estimation. Subsequently, the robotic arm was controlled to grasp the objects. As the gripper opening was much larger than the part diameter, it could tolerate certain estimation errors. After grasping, multiple-view data collection of the part was carried out, and the SCMV algorithm was used for pose re-estimation. The number of multi-views collected was set at 15 based on the SCMV algorithm. The captured multi-view images are shown in Figure 6.

The 15 sets of data were optimized using the SCMV algorithm to obtain the final pose of the object relative to the robotic manipulator end effector. By using the teach pendant of AUBO-i5, we can obtain the pose of the end effector, which is considered as the ground truth. Therefore, the error of our SCMV pose re-estimation algorithm could be calculated, as shown in Table 3.

**Table 3:** Pose of the object part relative to the robotic manipulator end effector and its error.

	**Pose of the parts**						**Error**	
	*x*(*m*)	*y*(*m*)	*z*(*m*)	α(rad)	β(rad)	γ(rad)	dt(mm)	*dr*(*rad*)
Part 1	0.0104	−0.0085	0.1060	1.6791	−0.4359	1.6281	1.469	0.039
Part 4	0.0138	−0.0043	0.1009	1.4367	−0.3961	1.6281	2.739	0.023
Part 5	0.0126	−0.0067	0.1096	1.5369	−0.5357	1.6281	2.569	0.041

It is not difficult to see from the table that the accuracy of the pose estimation is very high. After obtaining the final pose of the object relative to the robotic arm end effector, considering the high control accuracy of the robotic arm during movement and that the gripper holds the object without slipping, the part can be precisely controlled to perform any trajectory movement, thus completing subsequent assembly tasks.

### 4.5. Applications of peg-in-hole assembly

In this section, the SCMV pose estimation algorithm is tested for peg-in-hole assembly on a real robotic arm equipped with an AUBO-i5 robotic arm, a parallel two-finger gripper, and a realsense L515 camera for RGB image acquisition.

**Figure 7:**
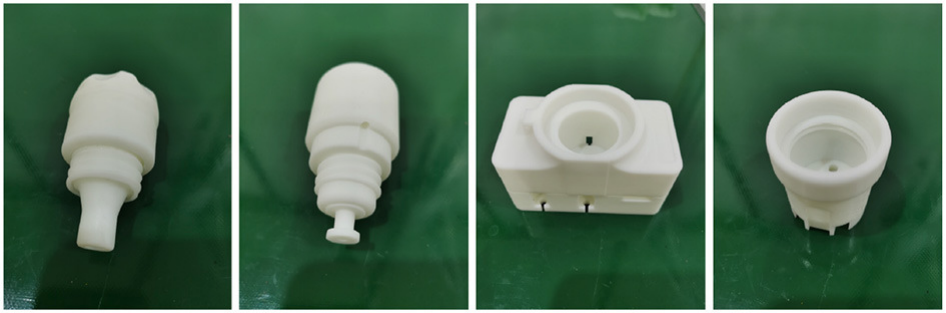
Diagram of parts to be assembled, from **left** to **right**, parts No.1, No.2, No.5, and No.19.

We use T-LESS industrial parts as parts to be assembled for assembly tasks. For actual assembly experiments, we utilized the 3D-printed parts model. The 3D printing materials used in these models are R4600 resins. With the SLA process, the accuracy can reach 0.2 mm, and the model has high flexibility, good size stability, and is suitable for assembly tasks. The diagram of the actual model of the part to be assembled is shown in Figure 7.

**Figure 8:**
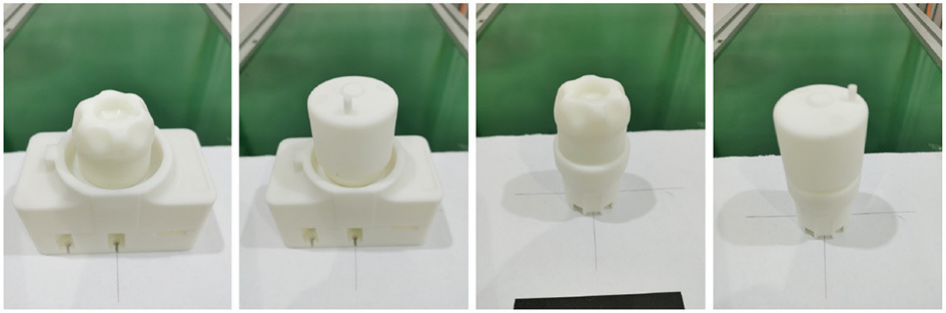
Diagram of assembly process (partial parts).

These parts include convex parts and concave parts, and when combined, four sets of bore assemblies can be obtained, as shown in Figure 8.

For this experiment, the task was simplified by fixing one part while using vision to detect and manipulate the other part with the robot arm to complete the assembly task. The fixed part's relative pose was obtained by the QR code visual positioning method, and its position was measured with a straightedge to ensure that it remained fixed in place. However, due to manual intervention, there may be an error of approximately 1mm in the fixed part's position.

**Figure 9:**
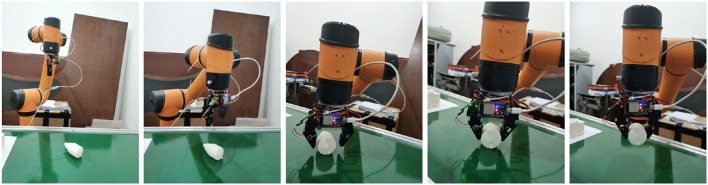
Schematic diagram of fixed assembled body parts.

The first step in the assembly system process is the grasping process, as shown in Figure 9.

**Figure 10:**
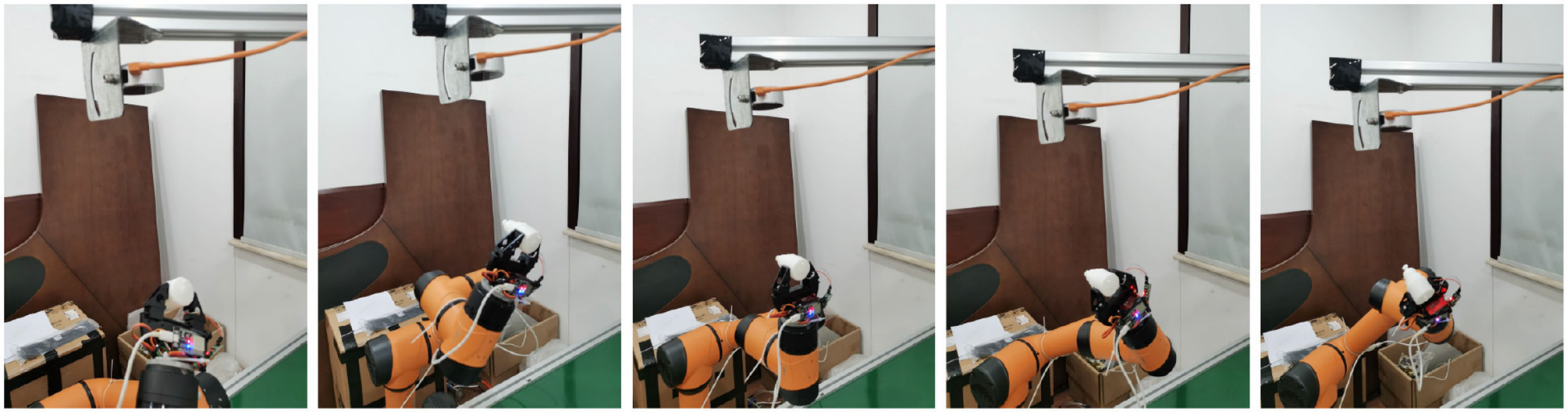
SCMV 6DoF pose estimation.

The second step in the assembly task is the pose SCMV 6DoF pose estimation, as shown in Figure 10. After the parts are grasped, the assembly parts need to be moved to the revaluation center first. The robot arm is then controlled to move in sequence based on the predetermined views, and the camera is used to capture images and obtain multi-view data. After the collection is completed, the relative position between the parts and the end-effector is estimated using the SCMV pose estimation algorithm.

**Figure 11:**
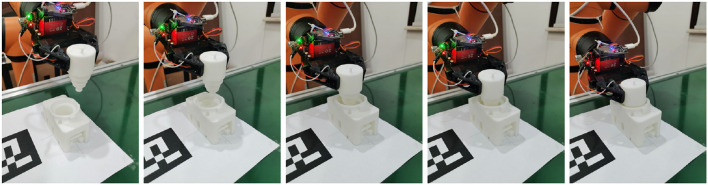
Parts assembly.

The third step in the assembly task is shown in Figure 11. After obtaining a relatively accurate pose of the part, it is firstly moved to the vicinity of the assembled body and then assembly path planning is carried out using the reinforcement learning model learned in the simulation environment. The assembly path sequence is then transformed into a joint trajectory sequence for the robot arm. The robot arm is controlled to move according to the joint trajectory sequence to complete the assembly task.

We test the entire system to evaluate the assembly success rate. For each assembly combination, we tested it 30 times. The test results are shown in Table 4. Over 80% of the grasping tasks are successful.

**Table 4:** Assembly experiment results.

	**Success times**	**Failed times**	**Success rate (%)**
Assembly combination 1	24	6	80
Assembly combination 2	27	3	90
Assembly combination 3	25	5	83
Assembly combination 4	24	6	80

We test the entire system to evaluate the assembly success rate of the system. For each assembly combination, 30 tests were conducted, and the reasons for failure were further refined into grasp failure and assembly failure. Grasp failure means that the object was not successfully grasped, while assembly failure indicates that the object was successfully grasped but clearly collided with the target during the final assembly stage. The test results are shown in Table 4.

It is easy to see from the table that some assembly combinations have a high success rate, with an assembly success rate of 90% for assembly combination 2, while the assembly success rate of some other combinations is not so high. The former has a relatively large margin of assembly and is relatively easy to assemble, while the latter is more prone to collisions during assembly due to accumulated errors during the assembly process, resulting in assembly failure.

The following are possible reasons for assembly failure:

1. For system calibration, the system calibration process differs between the simulation and real robot platform environments. In the simulation, both the pose of the robot manipulator and the camera can be directly obtained from the simulator, as well as the accurate intrinsics and extrinsics of the camera. Therefore, there are no calibration errors in the simulation environment. However, in the real robot platform, the pose of the robot manipulator in relation to the camera, the intrinsic matrix of the camera, and the extrinsics must be calibrated, resulting in potential calibration errors. The accuracy of the system calibration greatly affects the entire system's precision.
2. For a two-finger gripper, while a two-finger gripper can successfully grasp objects in simulation environments, there may be slight sliding when used in real-world scenarios, which can in turn affect the accuracy of pose re-estimation. In this paper's proposed SCMV algorithm, it is assumed that the gripper grasps the object and has no further relative motion with the assembly part. This condition is easily achieved during the simulation stage. However, in real-world applications, the contact surface between the parts and the gripper may be limited, resulting in insufficient contact between the fingers and the tips of the gripper. As a result, the robotic arm's abrupt stop motion during movement, due to inertia, could cause slight relative sliding or rotation of the parts, directly affecting the accuracy of pose re-estimation.

In this section, an intelligent assembly system based on the SCMV algorithm is designed, which can automatically perform part grasping and assembly tasks through monocular visual perception and reinforced learning planning. Finally, the functionality of the assembly system is verified through experiments, and a success rate of 90% is achieved for some assembly tasks. The reasons for the failure of some assembly tasks are also analyzed.

### 4.6. Error analysis of SCMV pose estimation

In this section, the single-view pose estimation is performed for each of the multi-views, and the error is computed to analyze the distribution of the pose estimation error for the various views. We conducted experiments with the T-LESS-GRASP-MV dataset, using the Cosypose single-view estimation algorithm for each view. If the estimation of translation error is less than 6 mm and the estimation of rotation error is less than 0.2 rad for each view, this view is deemed valid; otherwise, it is deemed invalid. The statistical results are shown in Table 5.

**Table 5:** Number of parts' valid views in the T-LESS-GRASP-MV dataset.

**Part number**	**No.1**	**No.4**	**No.5**	**No.6**	**No.11**	**No.13**	**No.19**	**No.20**	**No.21**	**No.23**
First grasp	30	41	44	42	31	35	28	41	14	40
Second grasp	15	34	44	38	29	18	43	38	12	39
Third grasp	18	45	44	43	26	29	19	30	18	36

**Figure 12:**
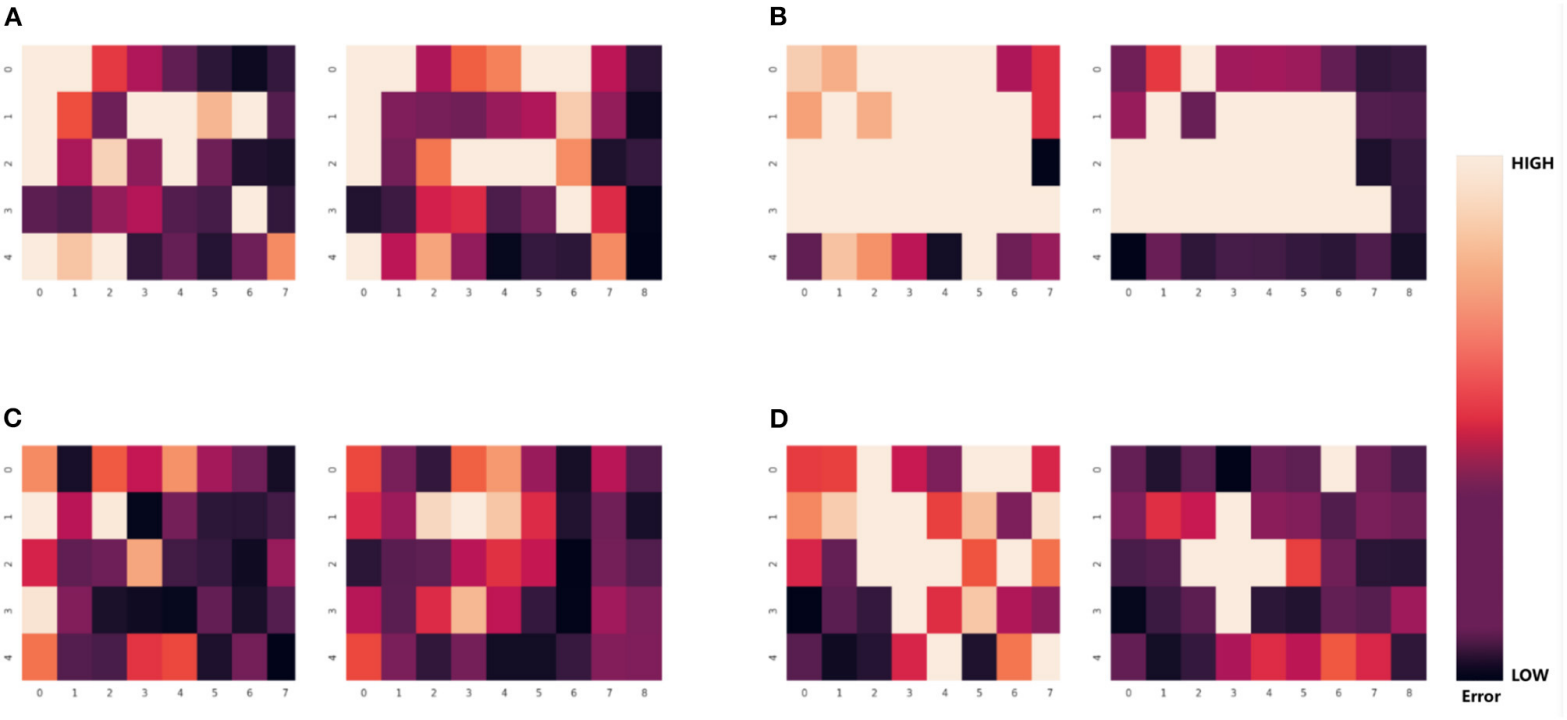
Error heatmap of the part in multiple views. Each large colored block in the heatmap represents the pose estimation error of 30 different viewpoints. Each small colored block represents the error between the result obtained by using single-viewpoint pose estimation method with front-view information and the true value. The darker the color, the lower the error. **(A)** The error of pose estimation in the first grasping for part No.1 in 30 viewpoints. **(B)** The error of pose estimation in the second grasping for part No.1. **(C)** The error of pose estimation in the first grasping for part No.4. **(D)** The error of pose estimation in the second grasping for part No.4.

It is evident from Table 5 that the number of valid views varies for various parts and even for the same part in various grasping poses. This result demonstrates that the errors in the pose estimation from different views are not independently and uniformly distributed but are instead related to the part's shape and occlusion. As shown in Figure 12, the error in pose estimation for all valid views of part 1 and part 2 is plotted as a heatmap in order to visualize the error distribution from different views.

For each of the four sets of results depicted in Figure 12, the left plot represents the translation error heatmap, and the right plot represents the rotation error heatmap, where the darker the color, the smaller the error. Nonetheless, for a particular part in a particular grasping pose, the error distribution may follow a particular pattern. For instance, in Figure 12B, the low-error views are concentrated at the edges, whereas the error is relatively greater for the views in the central region.

The SCMV pose estimation method automatically selects a set of better views and uses an optimal method to fuse multi-view information, avoiding the need to display the selection of the optimal view, which is more robust than directly selecting the optimal view.

## 5. Conclusion

A novel method that utilizes the initiative of robotic arm to obtain multi-view information of parts under single camera for 6DoF pose estimation is introduced. We first established an optimization model for the Single-camera Multi-view (SCMV) 6DoF pose estimation problem. Then, we refine the multi-view image sequence for the SCMV pose estimation method. We showed sampling the multi-view information with the initiative of robotic arm gained a superior performance. We also showed that refining the sequence of the multi-view image sequence, especially for the circumstances of the collision of the gripper with assembly parts and the gripper's self-occlusion while grasping, further improved pose estimation. We reported the outstanding performances on T-LESS-GRASP-MV datasets and demonstrated the robustness of the proposed approach on the real robot platform by successfully completing the peg-in-hole assembly task.

## Data availability statement

The raw data supporting the conclusions of this article will be made available by the authors, without undue reservation.

## Author contributions

ZG design the conceptualization. SY, ZG, and LY contributed to conception and design of the study. SY wrote the first draft of the manuscript. SY and ZG wrote sections of the manuscript. All authors contributed to manuscript revision, read, and approved the submitted version.
